# Supplementary material for: Identification of Functional Brassinosteroid Receptor Genes in Oaks and Functional Analysis of QmBRI1
Source: Int J Mol Sci. 2023 Nov 16;24(22):16405. doi: 10.3390/ijms242216405 (PMC10671120; doi:10.3390/ijms242216405)
Supplement: Supplementary file 1 [file ijms-24-16405-s001.zip › Supplementary Figure.pdf]

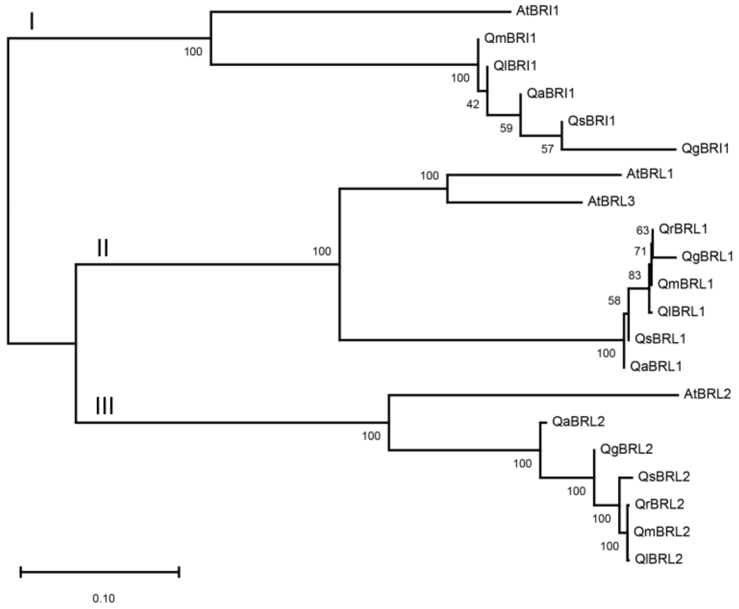

Figure S1. Phylogenetic tree of *BRI1-BRLs* genes identified in *Q. mongolica*, *Q. lobata*, *Q. suber*, *Q. gilva*, *Q. acutissima*, *Q. robur*, and *A. thaliana*. The Neighbor–Joining method was used to estimate evolutionary relationships with 1000 bootstrap replicates.

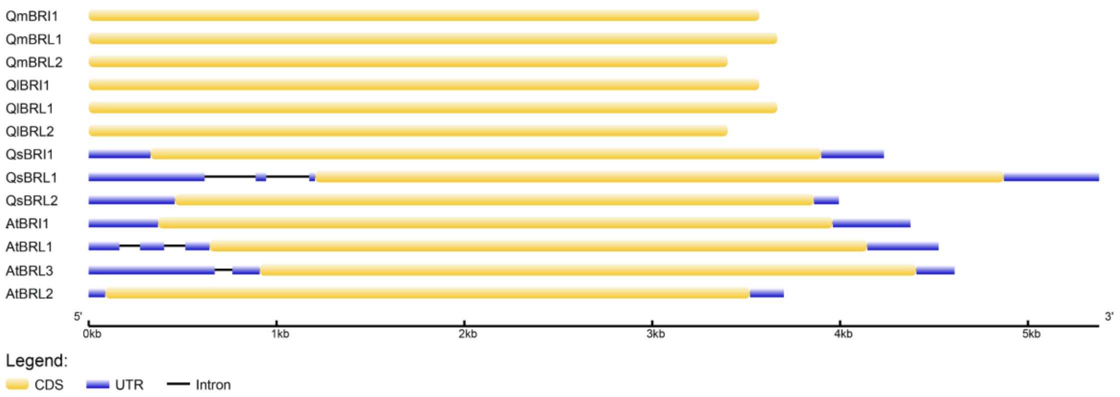

Figure S2. Gene structure analysis of *BRI1-BRLs* in *Q. mongolica*, *Q. loabta*, *Q. suber*, and *A. thaliana*.

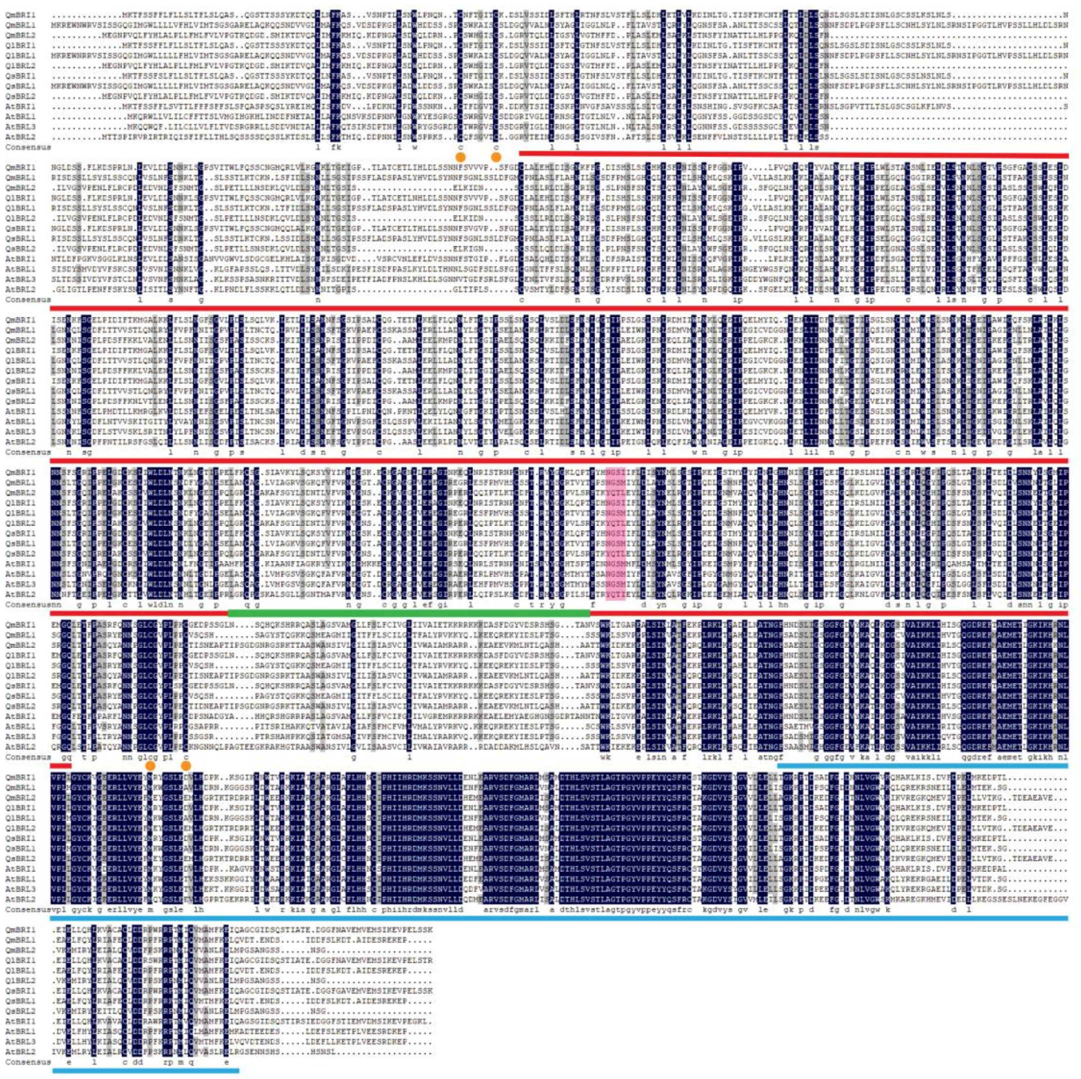

Figure S3. Protein sequence alignment of BRI1-BRLs in *Q. mongolica*, *Q. lobata*, *Q. suber*, and *A. thaliana*. Identical and similar amino acids are highlighted by dark blue and gray boxes, respectively. Orange dots indicate the paired cysteines flanking the LRR-domain; Red line, leucine-rich repeat (LRR) domain region; green line, the 70-amino acid island (ID) domain region; blue line, the serine/threonine (Ser/Thr) kinase domain region; pink region, the location of canonical Asn-Gly-Ser-Met (NGSM) motif.

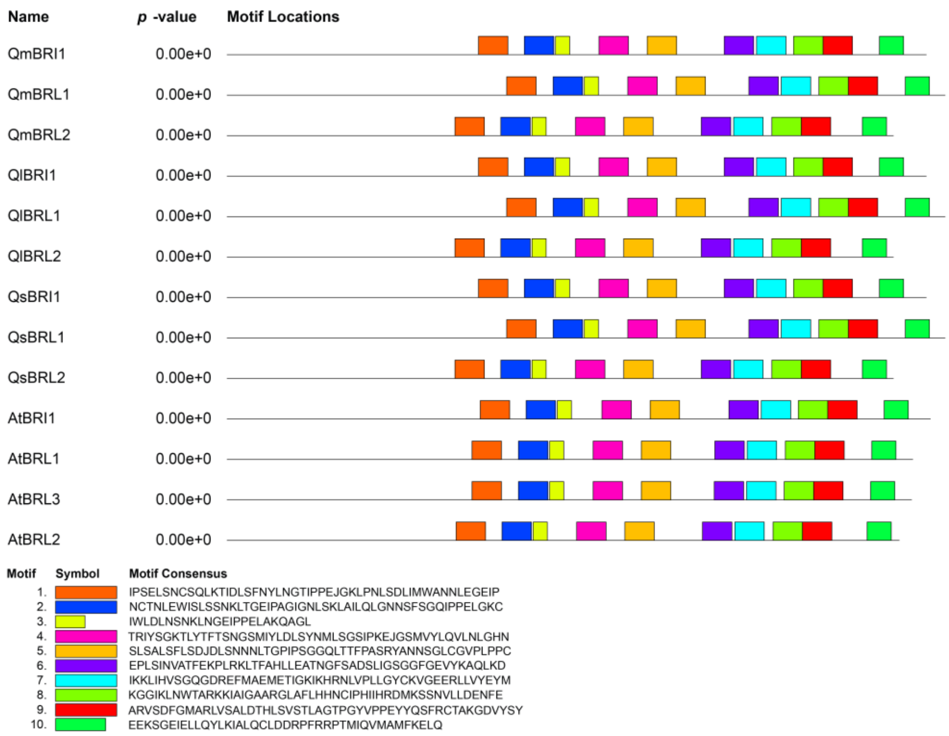

Figure S4. Motif characterization of BRI1-BRLs in *Q. mongolica*, *Q. loabta*, *Q. suber*, and *A. thaliana*.

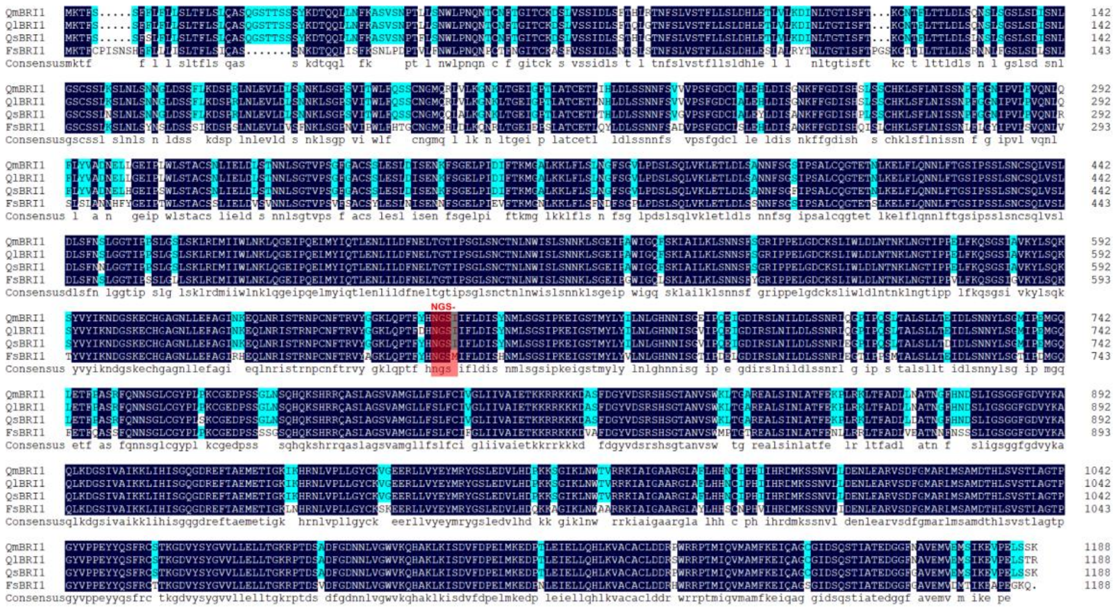

Figure S5. Protein sequence alignment of BRI1s in *Q. mongolica*, *Q. lobata*, *Q. suber*, and *F. sylvatica*. Identical and dissimilar amino acids are highlighted by dark blue and lake blue boxes, respectively. Red region, the location of canonical Asn-Gly-Ser-Met (NGSM) motif.

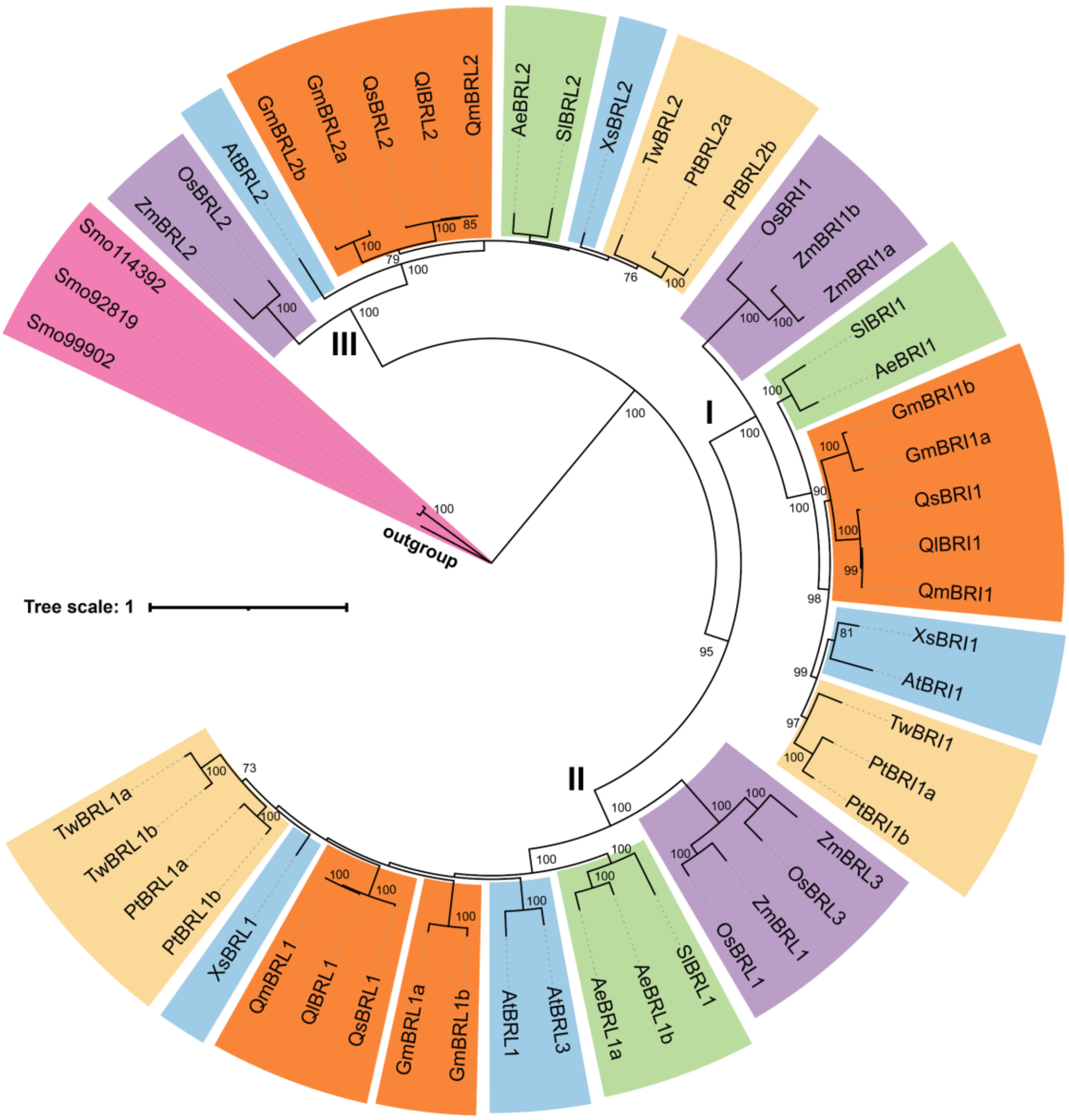

Figure S6. Phylogenetic tree was constructed using the Maximum Likelihood method based on the filtered multiple alignments. Homologs of EMS1 from *Selaginella moellendorffii* were used as an outgroup. Maximum Likelihood bootstrap value equal or greater than 70 % are given at the nodes.

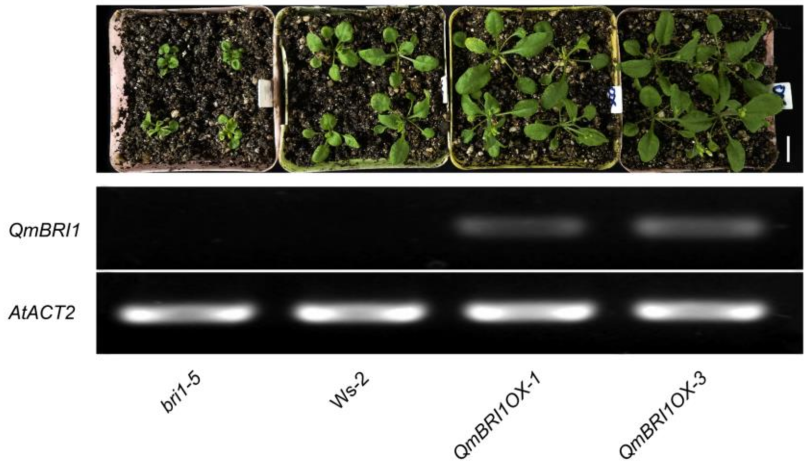

Figure S7. Phenotypic characterization and molecular identification of 3-week-old Arabidopsis lines. The expression of *QmBRI1* in the transgenic lines was verified by semi-quantitative analysis. The *Arabidopsis Actin 2* gene was used as an internal control.
